# Nonsyndromic Parkinson disease in a family with autosomal dominant optic atrophy due to *OPA1* mutations

**DOI:** 10.1212/NXG.0000000000000188

**Published:** 2017-09-22

**Authors:** David S. Lynch, Samantha H.Y. Loh, Jasmine Harley, Alastair J. Noyce, L. Miguel Martins, Nicholas W. Wood, Henry Houlden, Helene Plun-Favreau

**Affiliations:** From the Department of Molecular Neuroscience (D.S.L., J.H., N.W.W., H.H., H.P.-F.), UCL Institute of Neurology, London, UK; MRC Toxicology Unit (S.H.Y.L., L.M.M.), Leicester, UK; Reta Lila Weston Institute of Neurological Studies (A.J.N.), UCL Institute of Neurology, London, UK; and Neurogenetics Laboratory (H.H.), National Hospital for Neurology and Neurosurgery, London, UK.

Many genes implicated in familial Parkinson disease (PD) code for proteins with mitochondrial function.^1^ Several of these genes, including *PINK1* and *PARK2*, are involved in mitophagy, a mitochondrial quality control pathway.^2^ We describe a family with 3 members affected by autosomal dominant optic atrophy in which 2 affected members also developed PD.

While the role of mitophagy-related genes in PD is well established, this report provides further evidence of PD risk conferred through abnormal mitochondrial fusion and cristae morphology.

## Clinical description.

Patient 1 (P1) developed visual impairment in childhood. She was severely affected by optic atrophy but otherwise well until age 70 years. She then developed depression, followed by asymmetric upper limb tremor. This was accompanied by postural instability and a bradykinetic gait. She was diagnosed with PD and has a moderate response to levodopa. Patient 2 (P2) developed visual symptoms at age 9 years and was diagnosed with optic atrophy. Her vision deteriorated in her 20s, but she remained well until age 40 years when she developed gait difficulty and upper limb rest tremor. She was diagnosed with PD and has a moderate response to both levodopa and dopamine agonists. During examination at age 53 years, she had Hoehn and Yahr Stage 2 PD. Additional features, such as dementia, neuropathy, or deafness, were not present in P1 or P2. The youngest sibling, patient 3 (P3), was diagnosed with optic atrophy at age 14 years, and while visually impaired, remained mildly affected and can drive and read without magnification at age 40 years. She does not fulfill clinical diagnostic criteria for PD at this time. See figure, A for pedigree.

**Figure:**
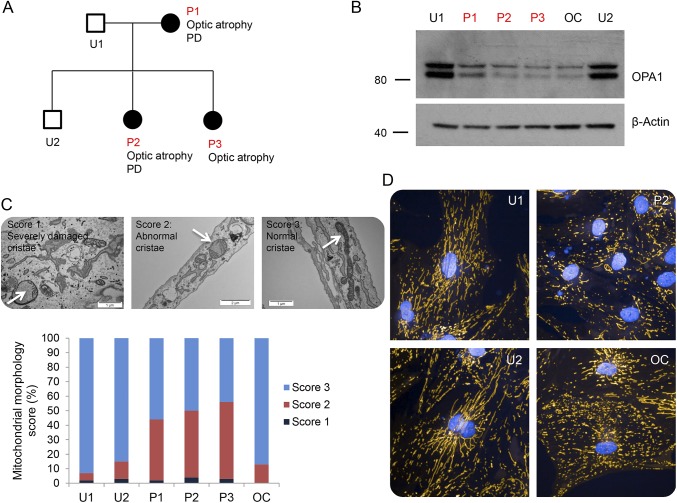
Pedigree, immunoblot mitochondrial morphology score, and confocal microscopy images of mitochondrial networks showing representative cells (A) Pedigree, in which filled boxes represent *OPA1* mutation carriers. (B) Immunoblot demonstrating reduced OPA1 protein levels in all affected patients. (C) Mitochondrial morphology score demonstrating a significant increase in the number of abnormal mitochondrial cristae found in patients P1, P2, and P3. Examples of each score are shown using white arrows. (D) Confocal microscopy images of mitochondrial networks showing representative cells. OPA1 patient cells showed an increased number of fragmented mitochondrial networks compared with controls. PD = Parkinson disease.

Both P2 and P3 were clinically assessed using a test battery used in the PREDICT-PD study, a population-based cohort in which participants are risk stratified for future PD using a variety of determinants.^3^ P2, who already carried a diagnosis of PD, scored 16/40 in the University of Pennsylvania Smell Identification Test (UPSIT), indicating anosmia. Her sister, P3, scored 33/40 in the UPSIT, which is within the normal range. A validated assessment of bradykinesia and incoordination, the BRAIN test, was performed by both P2 and P3. P2 scored in the low normal range for speed with a prolonged dwell time. She was also inaccurate and had poor rhythm. P3 scored in the normal range for speed, accuracy, and dwell time. These findings support the clinical observation that P3 does not fulfill motor criteria for PD, nor does she have objective olfactory or motor dysfunction that might suggest prodromal disease. She is, however, 10 years younger than her affected sister.

All 3 patients were found to carry the same heterozygous deletion of exons 28 and 29 of *OPA1*. We excluded point mutations in autosomal dominant PD genes via focused exome sequencing in patient P2 (Illumina TruSight One). Specifically, no rare variants were detected in *SNCA, LRRK2, GBA, PINK1, PARK2,* or *HTRA2*. An unrelated patient (OC) carrying a heterozygous frameshift mutation at the beginning of exon 28 (c.2708_2711 delTTAG) was used as a control. He was not affected with and had no family history of PD.

## Methods.

All 5 members of the family and patient OC underwent skin biopsy and dermal fibroblast culture. OPA1 protein levels were determined by Western blotting from whole cell lysates using the anti-OPA1 antibody (BD Biosciences), which recognizes both long and short isoforms of OPA1. Anti-β-actin was used as a loading control (Abcam, Cambridge, United Kingdom). Transmission electron microscopy (TEM) was used to investigate mitochondrial cristae morphology. Cells were fixed and imaged, and mitochondrial morphology was scored in a masked way in which a score of 3 indicated normal cristae morphology, a score of 2 indicated internal disorganization, and a score of 1 indicated mitochondria with severely damaged cristae.^4,5^ Mitochondrial networks were assessed in live cells after staining the mitochondria with tetramethylrhodamine and imaging at 40× under an Opera Phenix Microscope (Perkin Elmer, Waltham, MA).

## Results.

All affected patients showed an approximate 50% reduction in OPA1 levels by Western blotting (figure, B), in keeping with haploinsufficiency. Examination of mitochondrial morphology by TEM revealed a significant increase in abnormal cristae in P1, P2, and P3, compared with their unaffected family members (figure, C). This phenotype was not shared by the unaffected OPA1 patient OC, despite all affected patients showing similar disruption of the mitochondrial network in live cell imaging (figure, D).

## Discussion.

Mutations in a number of nuclear-encoded mitochondrial genes are known to cause PD, some of which lead to dysfunction of mitochondrial quality control (mitophagy). In 1 previous report, a form of syndromic PD with dementia, neuropathy, and deafness was associated with OPA1 mutations.^6^ Dysregulated mitophagy and mitochondrial networks have also been detected in OPA1 patient cells.^7^ This report further implicates OPA1 in nonsyndromic, idiopathic PD associated with abnormal cristae morphology and mitochondrial networks.

## References

[1] Klein C, Westenberger A Genetics of Parkinson's disease. Cold Spring Harb Perspect Med 2012;2:a008888. 22315721 10.1101/cshperspect.a008888 PMC3253033

[2] Deas E, Wood NW, Plun-Favreau H Mitophagy and Parkinson's disease: the PINK1-parkin link. Biochim Biophys Acta 2011;1813:623–633. 20736035 10.1016/j.bbamcr.2010.08.007 PMC3925795

[3] Noyce AJ, R'Bibo L, Peress L, et al PREDICT-PD: an online approach to prospectively identify risk indicators of Parkinson's disease. Mov Disord 2017;32:219–226. 28090684 10.1002/mds.26898 PMC5324558

[4] Tufi R, Gandhi S, de Castro IP, et al Enhancing nucleotide metabolism protects against mitochondrial dysfunction and neurodegeneration in a PINK1 model of Parkinson's disease. Nat Cell Biol 2014;16:1–12. 10.1038/ncb2901 PMC4199097 24441527

[5] Lehmann S, Loh SHY, Martins LM Enhancing NAD + salvage metabolism is neuroprotective in a PINK1 model of Parkinson's disease. Biol Open 2017;6:141–147. 28011627 10.1242/bio.022186 PMC5312101

[6] Carelli V, Musumeci O, Caporali L, et al Syndromic parkinsonism and dementia associated with OPA1 missense mutations. Ann Neurol 2015;78:21–38. 25820230 10.1002/ana.24410 PMC5008165

[7] Liao C, Ashley N, Diot A, et al Dysregulated mitophagy and mitochondrial organization in optic atrophy due to *OPA1* mutations. Neurology 2017;88:131–142. 27974645 10.1212/WNL.0000000000003491 PMC5224718

